# Hexagonal Multielectrode Design for Recording Local Electrograms With Superior Characteristics

**DOI:** 10.1016/j.jacep.2025.03.011

**Published:** 2025-05-14

**Authors:** Mark G. Hoogendijk, Ruben Coronel, Rishi Arora, Marion Kuiper, Natasja de Groot, Rudolf A. de Boer, Edward J. Vigmond, Bastiaan J. Boukens

**Affiliations:** a Department of Cardiology, Cardiovascular Institute, Thorax Center, Erasmus MC, Rotterdam, the Netherlands; b Heart Center, Department of Clinical and Experimental Cardiology, Amsterdam Cardiovascular Sciences, Amsterdam University Center, Amsterdam, the Netherlands; c Department of Cardiology, University of Chicago, Chicago, Illinois, USA; d Department of Physiology, Cardiovascular Research Institute Maastricht, Maastricht University Medical Center, Maastricht, the Netherlands; e IHU Institut LIRYC, Fondation University Bordeaux, Talence, France; f Institute of Mathematics of Bordeaux, UMR 5251, University of Bordeaux, Talence, France; g Laboratory of Experimental Cardiology, Department of Cardiology, Leiden University Medical Center (LUMC), Leiden, the Netherlands.

**Keywords:** activation mapping, catheter, electrogram, multielectrode

## Abstract

Electrograms recorded with a local coaxial reference offer advantages over bipolar electrograms for activation mapping but require more electrodes. We demonstrate that these coaxial electrograms can be recorded with a triangular local reference that can be efficiently created using hexagonal multielectrode grids. Triangular and cross-shaped local references recorded with a custom multielectrode were virtually indistinguishable. In contrast to bipolar electrograms, the morphology of the coaxial electrograms was insensitive to electrode orientation relative to the activation front, and the activation time matched closely with local activation. Developing hexagonal multielectrode designs may improve the efficiency and precision of clinical activation mapping.

## CENTRAL ILLUSTRATION

Basic Electrode Configurations for Recording Coaxial Electrograms

Coaxial electrograms can be recorded more efficiently using triangular references that can scale up to hexagonal than with cross references in square multielectrode configurations. Potential future hexagonal multielectrode designs are depicted in the bottom part of the figure.

## Graphical abstract

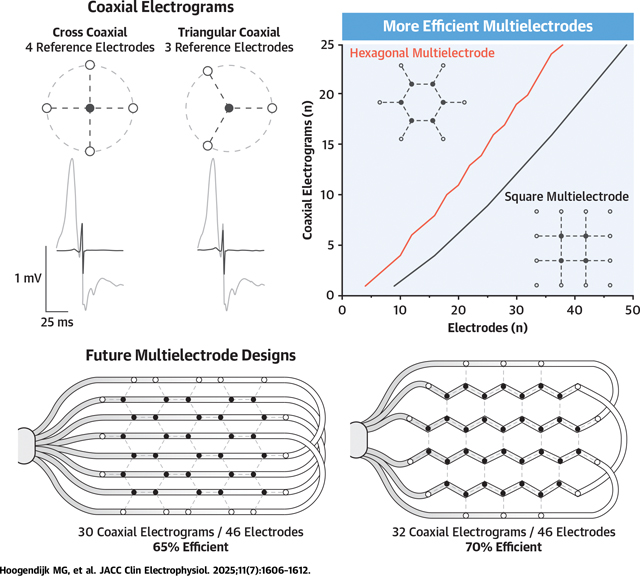

The presence of far-field signals and electromagnetic interference can hamper the precise annotation of activation in unipolar electrograms. Bipolar electrograms are often used during invasive electrophysiological procedures to reduce this interference.^1^ The use of bipolar electrograms, however, comes with disadvantages as their morphology and amplitude depend on the direction of the activation front relative to the electrode orientation.^2^ This complicates the identification of the precise local activation time. In addition, the localization of the activation signal is hindered by the dependence of bipolar electrograms on both contributing electrodes.

To overcome these drawbacks, the use of a local reference based on multiple electrodes has been proposed.^3–6^ When this local reference is created by averaging unipolar electrograms equally distributed around a recording electrode, they are called coaxial.^4^ Recently, Anter et al^5^ published a variation on this method in which a local reference was constructed based on a weighted contribution of surrounding electrograms depending on their similarity. The resulting local electrogram was termed multipolar. A disadvantage of the use of an evenly distributed group of surrounding electrodes is that these are not available at the edge of a multielectrode grid. This markedly reduces the number of electrodes that can serve as recording sites of coaxial and multipolar electrograms.

Therefore, we present a more efficient method to create a local reference using only 3 electrodes placed in an equilateral triangle around the recording electrode. At scale, these coaxial electrograms can be recorded using multielectrodes with a regular hexagonal electrode distribution.

## METHODS

### CUSTOM MULTIELECTRODE DESIGN.

We created a custom multielectrode to compare unipolar, bipolar, and coaxial electrograms with a cross and triangular local reference at the same location. The surface of the multielectrode was 3-dimensionally printed, after which 22 holes were drilled in a pattern based on a local overlapping of a square and regular hexagonal electrode distribution. Insulated silver wires (diameter 0.125 mm) were run through the holes, cut, and then polished, leaving the cross section of the silver wires exposed for recording. The interelectrode distance within the square and hexagonal electrode distributions was 1.2 mm.

### EXPERIMENTAL PREPARATION AND SETUP.

We used the porcine heart of an animal that was used for a different investigation, which was performed in agreement with national and institutional guidelines, and conforms with the European Commission Directive. It had been approved by the local Animal Experiments Committee, WP 2022–006-001. The anesthetized animal was euthanatized with pentobarbital. The explanted heart was perfused with a recirculating blood–Tyrode’s mixture that was gassed with 95% O_2_/5% CO_2_ and kept at a constant temperature in a Langendorff setup.

### DATA ACQUISITION.

A reference electrode was connected to the aortic root. The reference and multielectrode were connected to a BioSemi amplifier. Unipolar electrograms at each of the 22 electrodes were recorded at a sampling rate of 16 kHz. No filtering was applied.

After an equilibration period, the heart was defibrillated. Recordings were made from the left ventricular anterolateral and right ventricular outflow tract epicardium during spontaneous rhythm.

### DATA ANALYSIS.

Unipolar electrograms served as the basis for bipolar and coaxial electrograms with cross and triangular local references that were calculated using MATLAB R2021a software (Math-Works Inc). Bipolar electrograms were created by subtracting a neighboring unipolar electrogram from the recording electrode. Coaxial electrograms were recorded by subtracting a local reference based on the average of 4 neighboring unipolar electrograms in a cross or 3 in an equilateral triangular distribution surrounding a recording electrode. For comparing the activation time, we considered the −dV/dt_max_ in the unipolar electrogram the gold standard for the moment of local activation. Moment of maximal absolute voltage and −dV/dt_max_ were used as moment of activation in respectively the bipolar and coaxial electrograms.

### STATISTICS.

The comparison of amplitudes was conducted using a repeated measurement analysis of variance, whereas the comparison of local activation moments was based on an unpaired *t*-test (IBM SPSS Statistics for Macintosh). Data are shown as mean ± SEM. A *P* value of <0.05 was considered statistically significant.

## RESULTS

**FIGURE 1:**
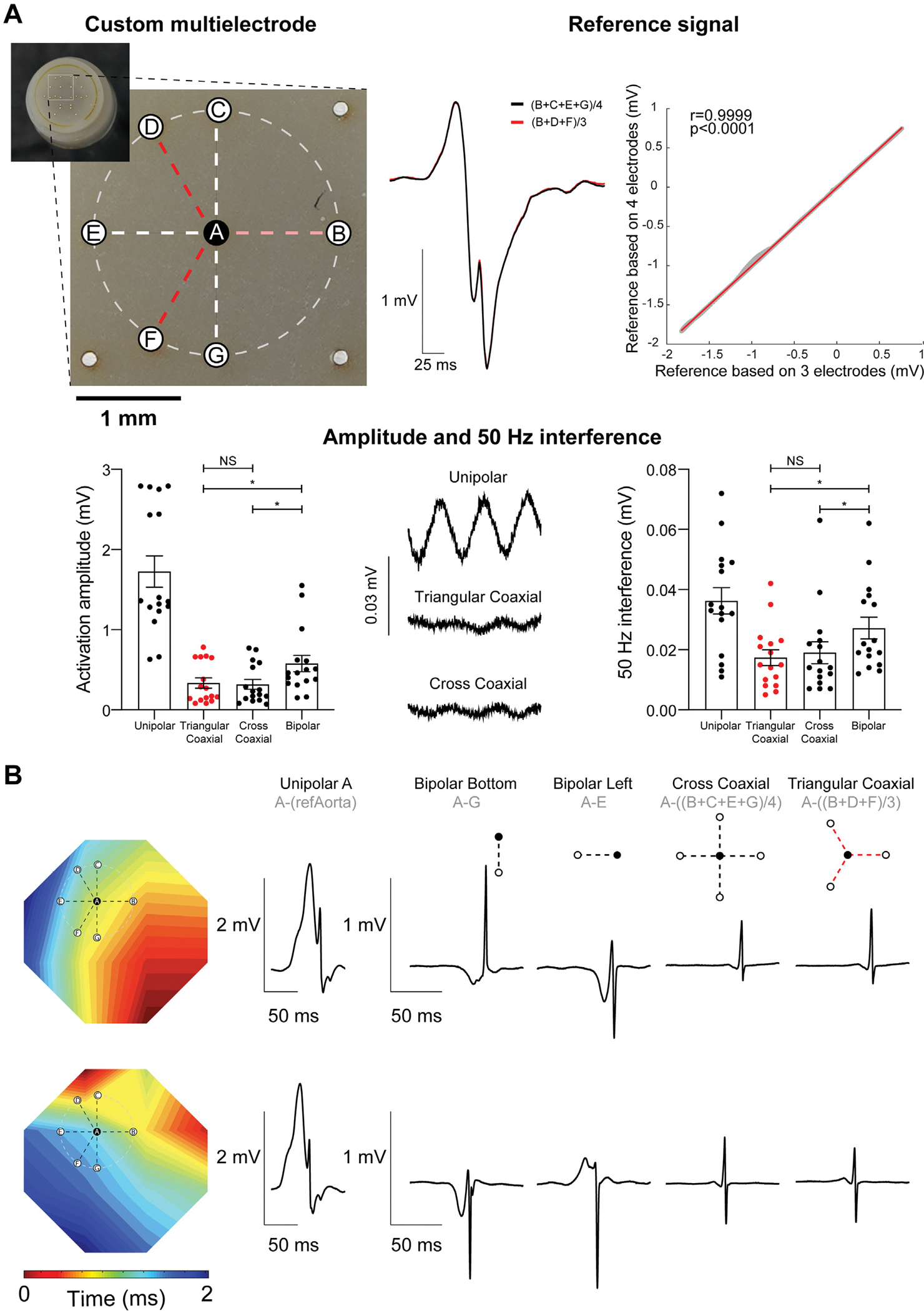
Feasibility Test Using a Custom Multielectrode Design (A) Depiction of the electrode distribution (left) and superimposed local reference electrograms using a cross and triangular reference and their correlation (right). The activation amplitude and 50 Hz interference of different types of electrograms are depicted in the middle. (B) Activation maps and unipolar, bipolar, and cross and triangular coaxial electrograms using the same recording electrode before and after rotation of the custom multielectrode are shown (below). *P < 0.05.

We first compared the local references to create the cross and triangular coaxial electrograms in recordings from the anterolateral left ventricle. These references showed an almost perfect match, supporting the feasibility of their use to record coaxial electrograms. The coaxial electrograms created with this reference contained the local contribution and retained the morphological features of the unipolar electrogram (Central Illustration). Also note the close similarities between both coaxial electrograms. We then compared the amplitude of the activation and 50 Hz interference in the recording preceding the activation in the different types of electrograms. In 4 recordings from the epicardial left anterolateral and 4 from the right ventricular outflow tract, we compared the amplitudes at the 2 available multielectrode sites for direct electrogram comparison. Both the amplitude of activation and 50 Hz interference were lower in cross and triangular coaxial than in unipolar or bipolar electrograms. (Figure 1A) The amplitude of activation relative to the 50 Hz interference did not differ between both types of coaxial and bipolar electrograms.

We then tested the effect of electrode orientation with respect to the activation front by rotating the custom multielectrode by approximately 90°. An activation map was made based on the local activation time of all 22 unipolar electrograms (Figure 1B). The activation front spread from right inferior to left superior before rotation of the electrode and from right superior to left inferior after. The unipolar, bipolar electrograms based on a local reference to the bottom and left, and coaxial electrogram based on a cross and triangular local reference are depicted before and after rotation of the multielectrode. As expected, altered multielectrode orientation towards the activation front had little effect on the local unipolar electrogram. It had a marked influence, however, on both the morphology and amplitude of the bipolar electrograms. The coaxial electrogram was insensitive to the altered electrode orientation relative to the direction of the activation front and, like the unipolar electrogram, maintained its morphology and amplitude.

**FIGURE 2:**
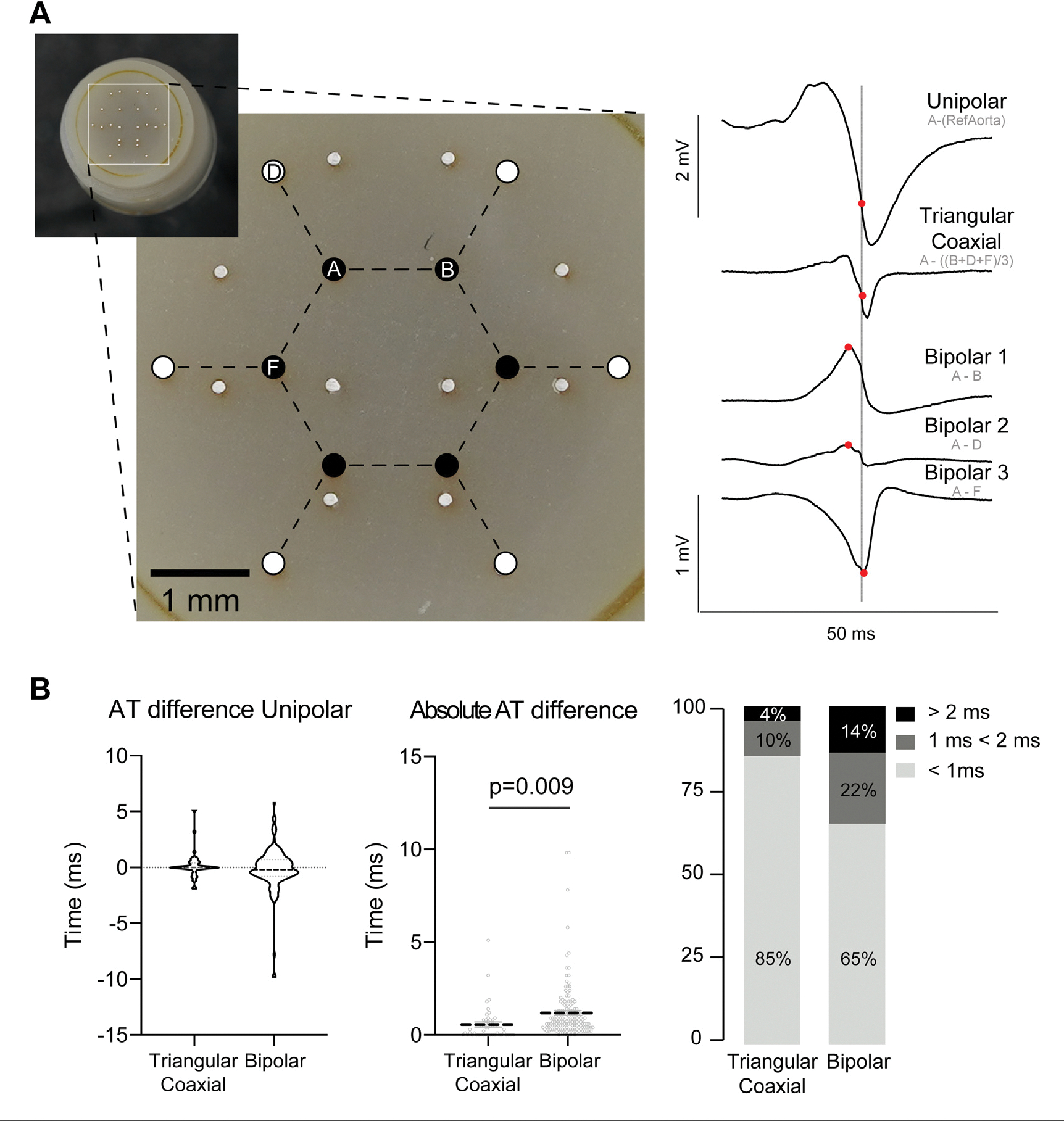
Comparison of Activation Times (A) Unipolar, triangular coaxial, and 3 bipolar electrograms recorded with a hexagonal multielectrode are depicted. The moment of activation is shown as a dot using the moment of activation on the unipolar electrogram as a standard (vertical line). (B) The difference in activation time between triangular coaxial or bipolar and unipolar electrograms.

Next, we compared the accuracy of activation mapping using either bipolar or triangular coaxial electrograms (Figure 2). For this, we made the same 8 ventricular recordings as for the amplitude testing. Twelve electrodes of the custom multielectrode that were part of the hexagonal pattern served as the basis to create unipolar, bipolar, and triangular coaxial electrograms. The moments of activation at the 6 central electrode locations (Figure 2A) were compared based on the unipolar electrogram, 3 bipolar electrograms, and the triangular coaxial electrodes using the same central electrode as recording electrode. The local activation matched better with the activation time in coaxial than bipolar electrograms with 46 of 48 (96%) vs 124 of 144 (86%) falling within 2 ms (Figure 2B).

## DISCUSSION

We present a first feasibility test of using coaxial electrograms based on a triangular local reference that can be recorded efficiently using a hexagonal electrode distribution. Further in vivo validation will be required before their use is considered during invasive electrophysiological procedures. Their characteristics, however, suggest their use may offer advantages over bipolar electrograms during activation mapping.

Our data show that the local activation mapping is more precise using coaxial instead of bipolar electrograms. Bipolar electrograms depict a regional voltage difference between 2 myocardial sites.^2^ Its morphology and amplitude, thereby, depend on the electrode orientation relative to the activation front.^7^ This dependence provides a challenge in determining the moment of activation in bipolar electrograms for which the maximal absolute voltage is normally used.^2^ Coaxial electrograms, on the other hand, are recorded using an evenly distributed reference that makes their morphology and amplitude independent of the electrode orientation relative to the activation front, and its moment of activation can be attributed to the central recording electrode.

A further advantage of coaxial electrograms is that they contain morphological information. In the early 2000s, Coronel et al^4^ already showed that Laplacian electrograms, which, like coaxial electrograms, are recorded using an evenly distributed local reference, can be used to identify the position within the myocardial activation being negative at the site of origin and positive at the latest activated myocardium. The morphological criteria of unipolar electrograms have been used to identify successful ablation sites of accessory bundles^8^ and premature ventricular complexes.^9^ The morphological information in the coaxial electrogram could take on a similar role as the unipolar electrogram, yet at a higher spatial resolution due to the shorter field of view.

Compared with other types of local electrograms, coaxial electrograms are most similar to multipolar electrograms.^5^ Multipolar electrograms are created using a local reference based on a weighted contribution of surrounding electrodes using a principal component analysis algorithm. In our study, we did not use such an algorithm, but calculated coaxial electrograms using a local reference based solely on the geometrical distribution of surrounding electrodes. Our data suggest that a geometrical solution based on an average of the 4 or 3 evenly spaced electrodes surrounding the recording electrode is effective in removing far-field components. This indicates that the use of a dedicated algorithm may not be required to remove remote components.

Omnipolar electrograms, on the other hand, appear to be less closely related to coaxial electrograms. Omnipolar electrograms are created by combining the information of 2 bipolar electrograms recorded in an orthogonal direction.^6^ The amplitude of omnipolar electrograms is, by design, independent on the direction of the activation front relative to the electrode orientation. The problem of the loss of morphological information of the unipolar electrogram and correct localization of activation to a single electrode, however, remains unresolved as omnipolar electrograms are essentially bipolar electrograms.

## CONCLUSIONS

Developing hexagonal multielectrode designs may improve the efficiency and precision of clinical activation mapping.

## PERSPECTIVES

### COMPETENCY IN MEDICAL KNOWLEDGE:

Coaxial electrograms can be recorded more efficiently using a hexagonal rather than a square electrode distribution.

### TRANSLATIONAL OUTLOOK:

Coaxial electrograms may improve the accuracy of activation mapping.
